# The impact of COVID-19 on sustainable development

**DOI:** 10.3325/cmj.2022.63.213

**Published:** 2022-06

**Authors:** Dragan Primorac, Richard J. Roberts

**Affiliations:** 1 St. Catherine Specialty Hospital, Zagreb, Croatia; 2 School of Medicine, University of Split, Split, Croatia; 3 University Department of Forensic Sciences, University of Split, Split, Croatia; 5 Faculty of Medicine, University of Osijek, Osijek, Croatia; 6 Faculty of Dental Medicine and Health, University of Osijek, Osijek, Croatia; 7 School of Medicine Rijeka, University of Rijeka, Rijeka, Croatia; 8 Eberly College of Science, Pennsylvania State University, University Park, PA, USA; 9 Henry C. Lee College of Criminal Justice and Forensic Sciences, University of New Haven, West Haven, CT, USA; 10 Medical School REGIOMED, Coburg, Germany; 11 The National Forensic Sciences University, Gandhinagar, Gujarat, India *dragan.primorac@svkatarina.hr*; 12 New England Biolabs, Ipswich, MA, USA; Both authors contributed equally to this work.

With more than 470 million confirmed cases of COVID-19 worldwide, and 6.1 million confirmed deaths (and perhaps three times that number, based on excess mortality as of February 27, 2022), the COVID-19 pandemic is the most important event of the 21st century so far (1,2). However, the number of people who recovered after COVID-19 as well as the newly developed vaccine have changed the course of the pandemic, and until now more than 10.8 billion vaccine doses have been administered (2).

The genome of the SARS-CoV-2 virus is composed of a single-stranded (positive-sense) RNA molecule and contains 29 903 nucleotides (3). After the first patients were reported in Wuhan, the first sequence of the virus (accession # LR757998) was released through the National Center for Biotechnology Information and European Bioinformatics Institute on January 30, 2020 (4), having been previously made public by Edward C. Holmes on January 11. Holmes, from the University of Sydney, was a member of a consortium led by Yong-Zhen Zhang of the Shanghai Public Health Clinical Center & School of Public Health, which published the sequence on https://virological.org/t/novel-2019-coronavirus-genome/319 as well as on GenBank (accession # MN908947). *The Lancet* quickly published an extensive genome analysis of the viruses extracted from nine patients. The sequences from the different patients matched at a percentage exceeding 99.98%, suggesting that the virus had only recently infiltrated the human population, most likely from a single location (5). Using a special spike-like glycoprotein on its surface, the SARS-CoV-2 virus binds to cells, mainly in the respiratory system, where it successfully uses the cell structures to produce a new generation of viruses. In the meantime, a number of scientists have shown that the so-called angiotensin-converting enzyme 2 (ACE2) receptors are key for virus entry into the cell (6). In addition to respiratory symptoms, COVID-19 patients were also diagnosed with multiple-organ involvement, including a deficiency of cellular immunity, coagulation activation, and myocardial damage, as well as liver and kidney damage (6). The increasing body of evidence suggests that chronic endothelial dysfunction is the crucial pathophysiological mechanism leading to a severe course of COVID-19 and worse patient outcomes. Cellular immunity plays an important role in people who recovered from SARS-Cov-2, presumably because that population group was exposed to several viral antigens, while in those who are vaccinated cellular immunity is created solely against the spike protein (7).

Currently, there is no way to precisely measure the total damage from the global SARS-CoV-2 pandemic. The socioeconomic impact of COVID-19 has varied among countries depending on surge inequalities, economic status, and vaccine availability when considered on a global scale. Two years ago, the COVID-19 outbreak disturbed educational systems worldwide. More recently, despite the Omicron variant, educational processes at all levels are operational and supported by safety protocols and vaccination programs. However, preliminary data suggest that the impact on the worldwide economy will be a GDP loss of at least 3.4% or approximately almost 2.96 trillion USD (8).

## How the virulence of the virus brought a rapid need for change

The incredible virulence of SARS-CoV-2 has been illustrated by Manski et al (9), who found a 10-fold increase in the probability of being tested and testing positive comparing New York and Italy between March 2020 and April 2020. Structural differences in viral variants have changed the binding affinity to the ACE2 receptors (10) and probably explain why this pandemic moved faster than those of previous iterations. This is further evidenced by increased Omicron affinity to the ACE2 receptor, caused by mutations in the receptor-binding domain (11). The rapid spread of the pandemic was compounded by an inappropriate perceived threat of the disease, which led many people to forego preventative measures such as masking. For example, one quarter of Italian health care workers did not view personal protective equipment as important (12). As such, this fast-paced pandemic with constantly mutating strains has proven difficult to contain, not only because of its virulence but also by the differential handling of it by the health care systems. The needs of society brought forth during the pandemic have resulted in tremendous steps forward by digitally transforming the distribution of goods throughout the service sector. There were also excellent examples of how society can move more efficiently toward sustainable development.

Following a good initial response by health care systems around the world, most countries’ public health institutions came alive with educational public health campaigns. Many countries put into place recommendations and restrictions that were aimed at slowing the spread of infection. The Netherlands (13) provided excellent example of how effective public health campaigns via social media targeting could result in improved personal hygiene.

## Changes that came about due to the availability of technology

This new-age approach to public health has also led to digital transformation and innovation in the technology sector. Restrictions that meant working from home for a lot of the general population created the perfect catalyst for the expansion of services such as Zoom and Microsoft Teams. In June 2019, Microsoft Teams boasted 13 million users (14), while in the second quarter of 2021 this number exponentially increased to 145 million active users (15). The Zoom meeting platform experienced a similar rise in popularity, with 10 million daily meeting participants in 2019 and as many as 350 million participants in December 2020 (16). These are phenomenal leaps responding to the many customers who wanted the same services while simultaneously wanting limited contact with the service providers. The COVID-19 pandemic has expedited the digitization of goods and services (17). The total effect is a time shift of three years globally, most notably in the Asia-Pacific markets (17). The knock-on effects of digital transformation can even be seen in the developing economies of Africa, which have several financial technology start-ups valued at over 1 billion USD. However, a severe hurdle to overcome in digital transformation is in the rural communities, which show internet usage to be only 27%, compared with 47% in urban areas (18). This illustrates the great need for further development in the provision of digital services to combat economic inequalities.

## Sustaining development for the future

We do not know if climate change will affect the transmission of SARS-CoV-2, but it is clear that climate change alters how we interact with different species on Earth. Some even underline that the COVID-19 pandemic has had a significant impact on reducing carbon emissions; however, its impact on slowing climate change is at best only temporary. The United Nations secretary-general has stated that climate change is the greatest threat to human life (19). The plans set forth in the UN’s environment program will have them harness the power of digital transformation in a three-tier plan. Through a sustainable ecosystem for the planet, shifting market and customer behaviors, and digital literacy and innovation they hope to reduce carbon emissions and the use of natural resources (20). Using digital transformation, they plan to create a standardized review process to streamline inefficiencies that result in excess carbon emissions. The discrepancies in gender equality, particularly those in developing countries (21), have to be taken into account to avoid unnecessary complications of such a tremendous step in the technology sector. The sustainability of the planet for future generations is a top priority, one which we are now better prepared to deal with inadvertently thanks to the COVID-19 pandemic.

## Role of technology in the COVID-19 pandemic

Challenging times are always an opportunity for technological progress. Technology has unquestionably helped control the pandemic, but also facilitated the continuation of life, activities, jobs, and medical care during lockdown and periods of isolation.

At the beginning of the COVID-19 pandemic, when there were no vaccines to prevent infection and no treatment protocols to help fight the disease, basic measures such as contact tracing, quarantine, masking, and lockdown were the only methods to control the pandemic. Today, despite vaccines and greater knowledge about the disease itself, these measures still remain the first line of defense against a pandemic in many countries around the world.

An important early success was the introduction of tests, developed through biotechnology, that enabled the rapid identification of infected individuals. As early as February 2020, loop-mediated amplification (LAMP) tests were implemented and tested in China (22). Later, polymerase chain reaction (PCR)-based tests have become the gold standard, despite the fact that LAMP-based tests are faster and do not require sophisticated instrumentation. Additional tests involving antigen detection are also now commonplace, including for use at home. However, the false-positive results obtained when using real-time reverse transcription-PCR (RT-PCR) tests to detect viral RNA, as well as the number of cycles required to reach the “cycle threshold,” have been discussed widely (23).

Although the pre-test measures had many shortcomings, digital technologies have helped in pandemic planning, surveillance, testing, contact tracing, quarantine, and health care. In some developed countries, technologies such as machine learning, artificial intelligence, real-time data from smartphones and wearable technologies (ie, smartwatches), thermal cameras, mobile phone applications, and web-based toolkits have been used for tracking, screening for infection, and contact tracing. Although advanced technologies can be expensive, require additional regulation and management, and can harm one's privacy, their use leads to faster and better isolation of infected individuals, provides a great deal of information about the disease and its prevalence, and slows the spread of infection, helping to maintain low mortality rates (24).

Digital technologies have also helped diagnose patients with severe symptoms of COVID-19. This is mostly seen in radiological diagnostics, where artificial intelligence and machine learning can differentiate COVID-19 from other lung diseases on CT scans and x-ray images, or in predicting the development of more severe forms of the disease (25,26).

Biotechnology has enabled the rapid development of the COVID-19 vaccine. The most-used vaccines against COVID-19 in the world are vaccines based on RNA technology. They are the first such vaccines licensed for use in the prevention of infectious diseases, although the RNA technology on which these vaccines are based has actually been developed over the last 20 years, but with the main goal of treating genetic diseases and cancer (27).

One of the major problems during the pandemic, especially in developing countries, was the lack of ventilators for patients with severe COVID-19, which led to poorer outcomes and lower survival rates. Advances in ventilator systems have resulted in faster ventilator production at a lower cost and thus greater ventilator availability for patients with COVID-19 (28). During the pandemic, not only did new technologies come to the fore, but some existing ones were also significantly optimized. Perhaps the best example is modern communication technologies such as video calling, which have been used for functions such as conferences, online teaching, and telemedicine.

Telemedicine is certainly not a new term in medicine, however, there has never been so much need to solve cases without the patient coming to the clinic or hospital. In the past two years, countless patients could not have come to the scheduled examination due to measures of self-isolation, or coming to the examination itself has posed a risk to the patient or the physician. These situations have led to the rapid implementation of telemedicine in almost all branches of medicine. Talking to the patient, taking a medical history, and making a preliminary examination via video calls have become commonplace in many developed countries (29). Although it is impossible to see the patient fully through video, the technology has enabled useful contact between the patient and the doctor at times when a complete live examination was not possible.

Technology is advancing day by day, and in times of crisis such as the COVID-19 pandemic, advances in technology have allowed many activities to be kept afloat despite the many restrictions the pandemic brings. As a result, technological advances that came to the fore during the pandemic will continue to be used to improve education and medical care.

## Impact of COVID-19 on education

As well as all fields of life, the COVID-19 pandemic has greatly affected education. Whether it is primary and secondary school students or college students, COVID-19 has brought great changes, mostly negative, in the lives of young people.

At a time when a person, due to any contact with a SARS-CoV-2 infected individual, has to self-isolate, when often entire classes have to be quarantined due to the presence of an infected student or employee, the transition from in-person to online teaching has become commonplace.

Although today's technology makes this type of teaching easier and such teaching is a better option than the complete abolition of school and college, it brings many other potential problems. E-learning, as the only option during the peak of the pandemic, served a purpose for some subjects in schools such as language arts. However, for subjects like math and science, it was impossible to learn everything through video lessons, resulting in poorer knowledge acquisition and poorer exam grades (30).

One of the problems is that due to children not going to school, parents often need to stay at home so that the child is not alone, or hire a caretaker, which further complicates the financial situation for families. School is not only about gaining certain knowledge and qualities, but it is also about providing a safe environment for children while their parents work, about serving meals, socializing with peers, engaging in physical activity, and the acquisition of social and communication skills (30).

The lockdown profoundly affected the lives and education of college students. Even though some students could benefit from online learning, the lack of live classes and the lack of socializing with colleagues negatively affected most students' mental health, work habits, and physical activity (31).

The students who may have lost the most by switching to e-learning are medical students. Clinical rotations were stopped to prevent the spread of COVID-19 in hospitals. Since clinical exercises are the most important and an unavoidable part of medical education, medical students lost this irreplaceable experience and knowledge. This is acknowledged by the students themselves, who mostly believe that e-learning cannot replace learning by the patient's bed (32). Furthermore, students are concerned that they will not gather enough knowledge and that they will be unprepared to finish college and start practicing medicine (33).

Attempts to compensate schools and colleges with e-learning succeeded in the intention that students do not lose years of schooling, but there are gaps in knowledge in certain subjects through all levels of the institution, which can be seen especially in medicine. Medical students are the future of medicine and should be afforded the best possible education; video lectures and seminars will never be a substitute for learning in clinical rotations.

## The impact of COVID-19 on science and technology

The COVID-19 pandemic has resulted in various effects on socio-economic activities and caused ongoing changes in science, technology, and innovation (29). While the economic impact has mostly been negative, especially in developing countries that had slow access to both tests and vaccines, there have been some positive gains as seen by digital companies providing remote access and biotechnology companies providing tests and vaccines. As with every disaster, the challenges offer opportunities for innovation and novel responses. Biotechnology and digital providers have responded magnificently and seem likely to play key roles going forward, especially as new pandemics are likely to appear. The main downside has been that in some countries, social media has enabled the naysayers to deny that a pandemic exists. They have been quite successful in promoting anti-science views that are now having political consequences. This is an issue that scientists need to address urgently if progress is to continue.

Although it is difficult to predict the long-term effects of the COVID-19 pandemic on science, technology, and innovation, it is important to explore all possible trends on how the pandemic could affect development in terms of overall consumption, digital infrastructure, openness, inclusiveness, and global cooperation (34). During the pandemic, various virtual communication and conferencing tools enabled new forms of collaboration between scientists, knowledge sharing, and the possibility of virtual education (35).

A larger number of scientific conferences, training activities, and research collaborations took place virtually in an attempt to increase the productivity of science and innovation. Virtual conferences have provided several benefits, such as a more diverse audience than face-to-face meetings and reduced transaction costs, as well as reducing environmental pollution by reduced airline flights. Such virtual tools have enabled access to training for a wider audience, and show high flexibility, making training more compatible with work commitments. By pooling expertise among institutions and enabling students to participate remotely in training offered by partner institutions, virtual tools could facilitate advanced education (34). However, there has been a cost in creativity as most professionals acknowledge that new and unexpected ideas arise much more easily during in-person meetings. After 25 years of tradition, the 2021 iteration of the ISABS conference was skipped due to COVID-19. After all possible efforts made to hold “The 12th ISABS Conference on Forensic and Anthropologic Genetics and Mayo Clinic Lectures in Individualized Medicine,” in-person attendance will be possible in beautiful Dubrovnik during June of this year (36).

The development of rapid information transfer has made it possible in the event of another future pandemic to accelerate the automation and business adoption of other technologies and practices. Results from companies that have successfully responded to the crisis during the pandemic point to many technological capabilities that others do not have – above all, filling gaps in technological advances during the pandemic, using more advanced technologies, and speed in experimentation and innovation (34,37).

The pandemic has also stimulated investment in blockchain technologies to increase transparency and trust in supply chains. Furthermore, trade barriers and relocation of production to locations where work is less expensive can further contribute to facilitating the process (34). The COVID-19 pandemic has shown the value and importance of new and improved forms of scientific communication such as the amount of data published on preprint servers and the way it is reviewed on social media platforms before official peer review. In that way, manuscripts and data can be quickly viewed, edited, analyzed, and published (38).

This rapid implementation of open science initiatives and open databases during the COVID-19 pandemic, which includes platforms for sharing research data, open access to COVID-19-related publications, and early distribution of research manuscripts as preprints, has accelerated the spread and acceptance of open data in all scientific research areas (34,39). Developing and accelerating the dissemination of scientific knowledge improves transparency and collaboration, reduces the risk of research work duplication, and encourages research and innovation built on existing research databases (34).

## The impact of COVID-19 on emerging technologies in cell and gene therapy

Gene and cell therapies represent innovations for the treatment of severe and inherited diseases, such as cancer, as well as rare genetic disorders and metabolic diseases. The COVID-19 pandemic disrupted the production and delivery of treatments and suspended research and the clinical development of various therapeutic options that are crucial for the gene and cell therapy (40).

The pharmaceutical industry, along with other key economic sectors, was severely disrupted during the COVID-19 pandemic as a result of investment in vaccines and the development of drugs against the SARS-CoV-2 virus. Stopping the development of other drugs has been particularly detrimental to the gene and cell therapy industry due to its complexity in production, supply chains, significant cost, and clinical trials. Some gene therapy development companies have shifted to the development of mRNA vaccines with similar technology. On the other hand, the COVID-19 pandemic has provided great opportunities for the pharmaceutical industry, exemplified by the need to rapidly develop drugs and vaccines against COVID-19. Companies that have developed gene and cell therapy had extensive experience in molecular biology, cellular immunity, genomic technology, and the production of viral vectors. These characteristics put them at an advantage in the research and development of promising therapies for COVID-19 (41).

The COVID-19 pandemic led to an unprecedented situation that affected all aspects of life, leading to uncertainty before the companies published the results of phase III clinical trials for two different mRNA-based vaccines (42). Thus, COVID-19 vaccines became the first licensed drugs to prevent infectious diseases using mRNA technology, although RNA vaccines and vector vaccines against adenovirus have been developed for more than 20 years for use in cancer and diseases caused by genetic mutations. Vaccines against COVID-19 very effectively prevent the disease in a relatively short period, however, their safety and efficacy will need to be monitored in the following years (27).

On the other hand, the COVID-19 pandemic has produced research and development of other forms of cancer therapy around the world, such as chimeric antigen receptor (CAR) T-cell therapy. Available data on the impact of the COVID-19 pandemic-related constraints on CAR T-cell delivery indicate the influence of pandemic waves and complement data from the biopharmaceutical industry highlighting the pandemic’s impact on cell and gene therapy detection and the delivery of licensed products (43). Nevertheless, some of the newer molecular technologies such as the rapidly advancing CRISPR technology may aid in tackling a global pandemic. During the COVID-19 pandemic, an urgent response was required to develop rapid and effective testing and diagnostic tools as soon as possible, alongside treatment options for patients with COVID-19 infection. Recently developed COVID-19 CRISPR Cas12-based test called SARS-CoV-2 DETECTR shows a short time of about 40 minutes to obtain results and an accuracy of 95%. In addition to CRISPR's molecular technology, CRISPR is potentially a tool for developing therapeutic options for patients with COVID-19. A Cas13 RNA-guided RNA-targeted endonuclease has recently been discovered and may serve as a potential therapeutic agent against COVID-19 (44).

In addition, successful treatment with mesenchymal stem cell therapy of patients with a severe clinical picture of COVID-19 has been demonstrated during the pandemic (45). Thus, the COVID-19 crisis has led to the accelerated and necessary use of the latest cellular and gene therapies, whose effectiveness and safety have yet to be clarified.

## Concluding remarks

The COVID-19 pandemic has demonstrated the previous fragility of our health care systems, which generally were unprepared for such a ferocious outbreak. However, it has also demonstrated the importance of biotechnology and digital technology in responding to the major challenge of a global pandemic. Thanks to previous developments in both fields, work, education, and daily living mostly managed to survive this pandemic and showed how important previous research was in providing the necessary tools, such as rapid diagnostic testing and vaccine development as well as remote video communication. In all three cases, the newly developed technologies promise that future responses to pandemics will likely be much more impactful than they had been previously. The pandemic also highlighted the shortcomings in response such as the inequality between urban and rural areas and between the developed world and the developing world. These issues will need to be addressed if we are to ensure a sustainable future for the planet. Even though it became the subject of interest for many, in this article we did not discuss the impact of different complications after recovering from COVID-19 or different sequelae after COVID-19 vaccination, simply because we still do not have relevant studies that gave clear answers to these questions.

In addition to health and communication issues, the availability of food in both developed and developing countries showed a sharp contrast and raised awareness of the need to do much better. Developing countries, in particular, need greater improvements in agricultural practices to ensure that populations can obtain sufficient nutritious food to respond both to pandemics and to the basic needs of life. It is time to recognize the value of crops that have been improved by biotechnology, so-called genetically modified crops. Science has demonstrated that such biotechnologically-improved crops are safe and do not need the draconian regulations that are preventing their widespread use.

Surely, by now we have learnt that politicians must respond to the needs of the world population and not to local self-interest. We all came together to fight the pandemic. Let us do the same to ensure a sustainable future for the planet.

## References

[1] Excess mortality during the Coronavirus pandemic (COVID-19) – our world in data. Available from: https://ourworldindata.org/excess-mortality-covid#citation. Accessed: March 19, 2022.

[2] COVID-19 United States Cases by County - Johns Hopkins Coronavirus Resource CenterAvailable from: https://coronavirus.jhu.edu/us-map. Accessed: February 27, 2022.

[3] Wu F Zhao S Yu B Chen YM Wang W Song ZG A new coronavirus associated with human respiratory disease in China. Nature 2020 579 265 9 10.1038/s41586-020-2008-3 32015508 PMC7094943

[4] Severe acute respiratory syndrome coronavirus 2 genome assembly, chrom - Nucleotide - NCBI. Available from: https://www.ncbi.nlm.nih.gov/nuccore/LR757998*.* Accessed: March 19, 2022.

[5] Lu R Zhao X Li J Niu P Yang B Wu H Genomic characterisation and epidemiology of 2019 novel coronavirus: implications for virus origins and receptor binding. Lancet 2020 395 565 74 10.1016/S0140-6736(20)30251-8 32007145 PMC7159086

[6] Šikić J Planinić Z Matišić V Friščić T Molnar V Jagačić D COVID-19: The impact on cardiovascular system. Biomedicines 2021 9 10.3390/biomedicines9111691 34829920 PMC8615470

[7] Primorac D Brlek P Matišić V Molnar V Vrdoljak K Cellular Immunity – The key to long-term protection in individuals recovered from SARS-CoV-2 and after vaccination. Vaccines (Basel) 2022 1 9 10.3390/vaccines10030442 35335076 PMC8953558

[8] Impact of the coronavirus pandemic on the global economy - Statistics & Facts | Statista. Available from: https://www.statista.com/topics/6139/covid-19-impact-on-the-global-economy/#dossierKeyfigures. Accessed: February 27, 2022.

[9] Manski CF Molinari F Estimating the COVID-19 infection rate: Anatomy of an inference problem. J Econom 2021 220 181 92 10.1016/j.jeconom.2020.04.041 32377030 PMC7200382

[10] Wan Y, Shang J, Graham R, Baric RS, Li F. Receptor recognition by the novel coronavirus from Wuhan: an analysis based on decade-long structural studies of SARS coronavirus. J Virol. 2020 Mar 17;94(7):1–9. 10.1128/JVI.00127-20 PMC7081895 31996437

[11] Kumar S Thambiraja TS Karuppanan K Subramaniam G Omicron and Delta variant of SARS-CoV-2: A comparative computational study of spike protein. J Med Virol 2022 94 1641 9 10.1002/jmv.27526 34914115

[12] Riccò M Vezzosi L Balzarini F Bragazzi NL Inappropriate risk perception for SARS-CoV-2 infection among italian HCWs in the eve of COVID-19 pandemic. Acta Biomed 2020 91 1 2 32921735 10.23750/abm.v91i3.9727 PMC7717016

[13] Yousuf H Corbin J Sweep G Hofstra M Scherder E van Gorp E Association of a public health campaign about coronavirus disease 2019 promoted by news media and a social influencer with self-reported personal hygiene and physical distancing in the Netherlands. JAMA Netw Open 2020 3 e2014323 10.1001/jamanetworkopen.2020.14323 32639569 PMC7344381

[14] Microsoft Teams reaches 13 million daily active users, introduces 4 new ways for teams to work better together. *Available from**:* https://www.microsoft.com/en-us/microsoft-365/blog/2019/07/11/microsoft-teams-reaches-13-million-daily-active-users-introduces-4-new-ways-for-teams-to-work-better-together/*.* Accessed: February 17, 2022.

[15] Microsoft Teams daily active users worldwide 2021 | Statista. Available from: https://www.statista.com/statistics/1033742/worldwide-microsoft-teams-daily-and-monthly-users/*.* Accessed: February 17, 2022.

[16] Zoom daily meeting participants worldwide 2020 | Statista. Available from: https://www.statista.com/statistics/1253972/zoom-daily-meeting-participants-global/. Accessed: February 17, 2022.

[17] COVID-19 digital transformation & technology | McKinsey. Available from: https://www.mckinsey.com/business-functions/strategy-and-corporate-finance/our-insights/how-covid-19-has-pushed-companies-over-the-technology-tipping-point-and-transformed-business-forever*.* Accessed: February 27, 2022.

[18] Executive summary | Africa’s Development Dynamics 2021: Digital Transformation for Quality Jobs | OECD iLibrary. Available from: https://www.oecd-ilibrary.org/sites/0a5c9314-en/index.html?itemId=/content/publication/0a5c9314-en. Accessed: February 17, 2022.

[19] Climate change: An “existential threat” to humanity, UN chief warns global summit. Available from: https://news.un.org/en/story/2018/05/1009782. Accessed: February 17, 2022.

[20] Digital TransformationAvailable from: https://www.unep.org/explore-topics/technology/what-we-do/digital-transformation. Accessed: February 17, 2022.

[21] Mariscal J Mayne G Aneja U Sorgner A Bridging the gender digital gap. Economics. 2019 13 2019 28 10.5018/economics-ejournal.ja.2019-9

[22] Zhang Y Odiwuor N Xiong J Sun L Nyaruaba RO Wei H Rapid molecular detection of SARS-CoV-2 (COVID-19) virus RNA using colorimetric LAMP. medRxiv. 2020;2:2020.02.26.20028373. 10.1101/2020.02.26.20028373

[23] Healy B Khan A Metezai H Blyth I Asad H The impact of false positive COVID-19 results in an area of low prevalence. Clin Med (Lond) 2021 21 e54 6 . Northfield Il 10.7861/clinmed.2020-0839 33243836 PMC7850182

[24] Whitelaw S Mamas MA Topol E Van Spall HGC Applications of digital technology in COVID-19 pandemic planning and response. Lancet Digit Health 2020 2 e435 40 10.1016/S2589-7500(20)30142-4 32835201 PMC7324092

[25] McCall B COVID-19 and artificial intelligence: protecting health-care workers and curbing the spread. Lancet Digit Health 2020 2 e166 7 10.1016/S2589-7500(20)30054-6 32289116 PMC7129544

[26] Wang L, Lin ZQ, Wong A. COVID-Net: a tailored deep convolutional neural network design for detection of COVID-19 cases from chest X-ray images. Sci Reports 2020 101. 2020 Nov 11;10(1):1–12. 10.1038/s41598-020-76550-z PMC7658227 33177550

[27] Nakagami H. Development of COVID-19 vaccines utilizing gene therapy technology.; Available from: https://academic.oup.com/intimm/article/33/10/521/6194108. Accessed: February 27, 2022. 10.1093/intimm/dxab013 PMC8083619 33772572

[28] Chow EKH Wong PK Ding X Advances in Technology to Address COVID-19. SLAS Technol 2020 25 511 2 10.1177/2472630320969634 33215941 PMC8960230

[29] Bokolo A Jr Use of telemedicine and virtual care for remote treatment in response to COVID-19 Pandemic. J Med Syst 2020 44 32542571 10.1007/s10916-020-01596-5 PMC7294764

[30] Hoofman J Secord E The Effect of COVID-19 on Education. Pediatr Clin North Am 2021 68 1071 9 10.1016/j.pcl.2021.05.009 34538299 PMC8445757

[31] Chaturvedi K Vishwakarma DK Singh N COVID-19 and its impact on education, social life and mental health of students: A survey. Child Youth Serv Rev 2021 ••• 121 10.1016/j.childyouth.2020.105866 33390636 PMC7762625

[32] Alsoufi A Alsuyihili A Msherghi A Elhadi A Atiyah H Ashini A Impact of the COVID-19 pandemic on medical education: Medical students’ knowledge, attitudes, and practices regarding electronic learning. PLoS One 2020 15 10.1371/journal.pone.0242905 33237962 PMC7688124

[33] Theoret C Ming X Our education, our concerns: The impact on medical student education of COVID-19. Med Educ 2020 54 591 2 10.1111/medu.14181 32310318 PMC7264564

[34] How will COVID-19 reshape science, technology and innovation? - OECD. Available from: https://read.oecd-ilibrary.org/view/?ref=1098_1098772-3qmm9rpta1&title=How-will-COVID-19-reshape-science-technology-and-innovation*.* Accessed: February 27, 2022.

[35] Paunov C and SPS. Science, technology and innovation in the time of COVID-19. OECD Sci Technol Ind Policy Pap. 2021;99.

[36] Vuk-Pavlović S Schanfield M Into the third decade: eleventh ISABS Conference on Forensic and Anthropological Genetics and Mayo Clinic Lectures in Individualized Medicine. Croat Med J 2019 60 189 90 10.3325/cmj.2019.60.189 31187945 PMC6563175

[37] Blackburn S, Laberge L, O’toole C, Schneider J. How COVID-19 has pushed companies over the technology tipping point—and transformed business forever. Available from: https://www.mckinsey.com/business-functions/strategy-and-corporate-finance/our-insights/how-covid-19-has-pushed-companies-over-the-technology-tipping-point-and-transformed-business-forever*.* Accessed: May 25, 2021.

[38] Aristovnik A Ravšelj D Umek L A Bibliometric analysis of COVID-19 across Science and social science research landscape. Sustain 2020 12 9132 10.3390/su12219132

[39] Why open science is critical to combatting COVID-19 - OECD. Available from: https://read.oecd-ilibrary.org/view/?ref=129_129916-31pgjnl6cb&title=Why-open-science-is-critical-to-combatting-COVID-19*.* Accessed: February 27, 2022.

[40] COVID-19 and cell and gene therapy | McKinsey. Available from: https://www.mckinsey.com/industries/life-sciences/our-insights/covid-19-and-cell-and-gene-therapy-how-to-keep-innovation-on-track. Accessed: February 27, 2022.

[41] Qiu T Wang Y Liang S Han R Toumi M The impact of COVID-19 on the cell and gene therapies industry: Disruptions, opportunities, and future prospects. Drug Discov Today 2021 26 2269 81 10.1016/j.drudis.2021.04.020 33892148 PMC8057929

[42] Abu Abed OS Gene therapy avenues and COVID-19 vaccines. Genes Immun 2021 22 120 4 10.1038/s41435-021-00136-6 34079091 PMC8170448

[43] Ghorashian S Malard F Yüksel MK Mauff K Hoogenboom JD Urbano-Ispizua A Defining the impact of SARS-COV-2 on delivery of CAR T-cell therapy in Europe: a retrospective survey from the CTIWP of the EBMT. Bone Marrow Transplant 2022 57 299 301 10.1038/s41409-021-01483-8 34802048 PMC8605455

[44] Uddin F Rudin CM Sen T CRISPR gene therapy: applications, limitations, and implications for the future. Front Oncol 2020 10 1387 10.3389/fonc.2020.01387 32850447 PMC7427626

[45] Primorac D Stojanović Stipić S Strbad M Girandon L Barlič A Frankić M Compassionate mesenchymal stem cell treatment in a severe COVID-19 patient: a case report. Croat Med J 2021 62 288 96 10.3325/cmj.2021.62.288 34212566 PMC8275939

